# Real-Time Study of Surface-Guided Nanowire Growth by *In Situ* Scanning Electron Microscopy

**DOI:** 10.1021/acsnano.2c07480

**Published:** 2022-10-28

**Authors:** Amnon Rothman, Kristýna Bukvišová, Noya Ruth Itzhak, Ifat Kaplan-Ashiri, Anna Eden Kossoy, Xiaomeng Sui, Libor Novák, Tomáš Šikola, Miroslav Kolíbal, Ernesto Joselevich

**Affiliations:** † Department of Molecular Chemistry and Materials Science, Weizmann Institute of Science, Rehovot76100, Israel; ‡ Institute of Physical Engineering, Brno University of Technology, Technická 2, 616 69Brno, Czech Republic; § CEITEC BUT, Brno University of Technology, Purkyňova 123, 612 00Brno, Czech Republic; ∥ Department of Chemical Research Support, Weizmann Institute of Science, Rehovot76100, Israel; ⊥ Thermo Fisher Scientific, Vlastimila Pecha 12, 627 00Brno, Czech Republic

**Keywords:** guided growth, planar nanowires, *in situ* growth, real-time monitoring, ZnSe

## Abstract

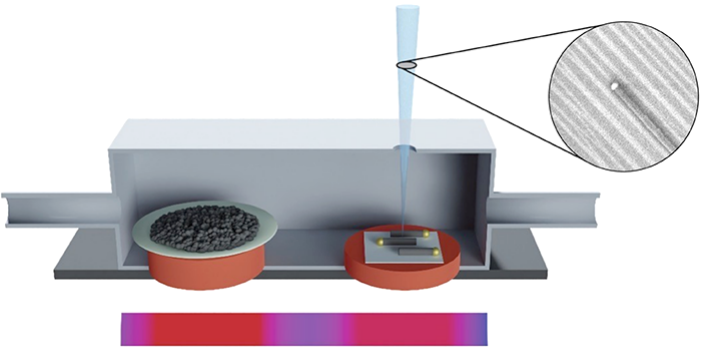

Surface-guided growth has proven to be an efficient approach for the production of nanowire arrays with controlled orientations and their large-scale integration into electronic and optoelectronic devices. Much has been learned about the different mechanisms of guided nanowire growth by epitaxy, graphoepitaxy, and artificial epitaxy. A model describing the kinetics of surface-guided nanowire growth has been recently reported. Yet, many aspects of the surface-guided growth process remain unclear due to a lack of its observation in real time. Here we observe how surface-guided nanowires grow in real time by *in situ* scanning electron microscopy (SEM). Movies of ZnSe surface-guided nanowires growing on periodically faceted substrates of annealed M-plane sapphire clearly show how the nanowires elongate along the substrate nanogrooves while pushing the catalytic Au nanodroplet forward at the tip of the nanowire. The movies reveal the timing between competing processes, such as planar vs nonplanar growth, catalyst-selective vapor–liquid–solid elongation vs nonselective vapor–solid thickening, and the effect of topographic discontinuities of the substrate on the growth direction, leading to the formation of kinks and loops. Contrary to some observations for nonplanar nanowire growth, planar nanowires are shown to elongate at a constant rate and not by jumps. A decrease in precursor concentration as it is consumed after long reaction time causes the nanowires to shrink back instead of growing, thus indicating that the process is reversible and takes place near equilibrium. This real-time study of surface-guided growth, enabled by *in situ* SEM, enables a better understanding of the formation of nanostructures on surfaces.

## Introduction

Single-crystal semiconductor nanowires (NWs) have attracted overwhelming attention over the last two decades owing to their physical properties, which make them promising building blocks for nanotechnology. Large-scale integration of the NWs into ordered arrays could lead to novel devices for a wide range of applications, as optoelectronics,^1,2^ logic circuits,^3,4^ and quantum computing.^5,6^ A common method to grow semiconductor NWs is the vapor–liquid–solid (VLS) mechanism, where a metal catalyst on the substrate surface forms a liquid alloy droplet with the semiconductor grown material and promotes the axial NW growth in the vapor phase.^7^ Guided growth is an attractive approach to integrate NWs into circuits and other planar devices, where the substrate surface directs the NW growth by three main modes:^8,9^ epitaxy, graphoepitaxy, and artificial epitaxy. In the epitaxial mode, the NWs grow along specific crystallographic directions dictated by epitaxial relations between the NW material and the substrate, usually a flat single crystal. In the graphoepitaxial mode, the NWs grow on a corrugated substrate along nanoscale topographic features, such as nanosteps or nanogrooves. In the artificial epitaxy mode, the NWs grow along artificially created guides, such as trenches and ridges that are lithographically patterned on an amorphous substrate or scratches created by mechanical polishing.^10^ Surface-guided NWs are a particular case of planar NWs (also referred to as “horizontal”, “in-plane”, or “lateral” NWs) where the NWs have well-defined directions determined by the substrate. Despite the technological potential of the surface-guided NWs, in order to gain prediction abilities and fully control this growth, a fundamental understanding of the mechanism of the guided nanowire growth is needed.

The surface-guided growth of in-plane NWs was demonstrated by Nikoobakht et al.^11^ and Li and co-workers^12^ for ZnO on A-plane sapphire and GaAs on GaAs, respectively. Joselevich and co-workers^9,13,14^ extended the surface-guided growth from epitaxial to graphoepitaxial GaN NWs on various planes of flat and faceted sapphire and later to other semiconductor materials, such as ZnO,^15,16^ ZnSe,^17^ ZnTe,^18^ CdSe,^19^ CdS,^20^ and ZnS,^21^ and other substrates, such as quartz,^22^ SiC,^13^ MgAl_2_O_4_,^14^ oxidized Si wafers,^8^ and glass.^10^ Heterostructures based on in-plane core–shell NWs were also demonstrated and integrated into photodetectors and photovoltaic cells.^23,24^ Surface-guided growth by non-VLS mechanisms has also gained ground in recent years, including surface-guided CsPbBr_3_ perovskite nanowire growth,^25−28^ selective-area growth of semiconductor NW networks on patterned single-crystal substrates,^29,30^ and NW growth by a solid–liquid–solid mechanism.^31,32^

The kinetics and mechanism of surface-guided NW growth were studied comprehensively only recently and revealed the effect of dimensionality on the diffusion transport of the semiconductor materials into the catalyst droplet.^33,34^ These studies present a highly predictive theoretical model of the planar growth kinetics that considers different pathways of material transport into the surface-guided NWs and shows that two main effects control the in-plane growth: the Gibbs–Thomson (GT) effect for thinner NWs and surface diffusion for thicker ones. These two opposed effects lead to a maximum NW growth rate at an optimal catalyst nanoparticle size and NW diameter. The model is manifested by the growth rate (d*L*/d*t*) dependence on the NW radius *R*, presented in eq 1, where *I* is the vapor flux of the elements, Ω is the elementary volume per pair of atoms in the solid, θ_lv_ and θ_ls_ are the ratios of liquid to vapor and surface to vapor activities, *R*_GT_ is the characteristic GT radius, λ is the effective diffusion length of the precursor adatoms, and *m* represents the dimensionality of the dominant surface diffusion pathway. According to the model, a value of *m* = 1 is expected when the main contribution originates from the surface diffusion of precursor adatoms along the NW sidewall, as it is the case for nonplanar growth. In surface-guided (i.e., planar) growth, where the catalyst is in contact with the substrate, the precursor adatoms can be collected directly from the substrate as well, and then *m* = 3/2 or *m* = 2, depending on the relation between the diffusion lengths on the NW sidewalls and on the substrate surface. This model was found to accurately fit the planar vs nonplanar NW growth kinetics.
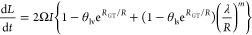
1

One serious limitation of these kinetic and mechanistic studies is that they are based on *ex situ* measurements of NWs *after* they have stopped growing, so they only provide indirect information on how the NW geometry evolves *during* their growth. The growth mechanism of the surface-guided NWs, however, involves many aspects that the conventional *ex situ* analysis techniques cannot tackle. Obtaining a comprehensive understanding of the growth mechanism requires observation of the growth process in real time. The current work presents a real-time study of surface-guided NW growth using *in situ* scanning electron microscopy (SEM). In this study, we grow ZnSe NWs along the nanogrooves of a periodically faceted surface (annealed M-plane sapphire, i.e., α-Al_2_O_3_ (1010), using a special heating stage inside an SEM, which allows us to directly watch NWs at nanometer-scale resolution as they grow. The recorded movies presented here show in great detail how guided NWs grow and reveal important aspects of the process that could not be observed in previous *ex situ* analyses, such as the instantaneous elongation velocity, the influence of surface defects on the growth direction, and the reversibility of the growth process.

Despite being known since 1964,^7^ the VLS mechanism for nonplanar NWs was explained in detail 30 years later, stemming from real-time *in situ* electron microscopy growth observation. Phenomena such as layer-by-layer growth,^35^ heterogeneous nucleation,^36^ diffusion of a metal catalyst into the NW,^37^ atomic step flow on a nanofacet,^38^ and the transport mechanism of the catalytic droplet^39^ were revealed, which significantly improved the current understanding of the growth mechanisms of these nanostructures.^40^ Most *in situ* studies were based on growth experiments of relatively well-known and straightforward materials, such as Si,^36,39^ Ge, or GaAs^40^ using Au as a catalyst, or others.^41−43^ Unlike the well-studied semiconductor NWs whose precursors are supplied in the gas phase and can be fed into a TEM chamber relatively easily, the precursors of other semiconductor materials, such as II–VI materials, chalcogenides, oxides, and part of the III–V materials, are in the solid phase. Moreover, in the case of in-plane NWs, TEM observation is not possible because the substrate is usually too thick and hence electronically opaque. Under such conditions, utilization of SEM or environmental SEM (ESEM) seems to be the only plausible option. However, such experiments are sparse, requiring more advanced precursor-delivery systems. Willinger and co-workers^44^ demonstrated the *in situ* observation of the growth kinetics of ZnS NWs using ESEM. They found that the growth rate of individual NWs correlates with the size of the metal catalyst at the tip. They also demonstrated an example of *in situ* catalyst splitting during the process of NW growth. Those kinetic events could not be precisely derived from postgrowth *ex situ* characterizations. Kolíbal et al.^45^ used a dedicated reactor chamber inside an SEM for real-time observation to elucidate the SiO_2_ NW growth mechanism with a Ga catalyst on a Si substrate. They showed that the presence of water or hydrogen has a critical effect on the morphology of the growth products due to the etching of a thin gallium oxide overlayer that was formed on the catalyst particles during sample preparation. Additional real-time observation of planar NW growth work was done by Cabarrocas and co-workers,^41−43^ showing a solid–liquid–solid growth mechanism, where the NW is formed by consuming and transforming surrounding a-Si:H into the crystalline NW. These studies do not involve a gaseous precursor.

Despite these numerous real-time studies of NW growth, real-time studies of surface-guided VLS growth of NWs by an *in situ* electron microscope have not been reported prior to the present work. Neither have movies of growing planar NWs been shown in previous reports. Although NW growth experiments in the electron microscopes are held under different conditions than conventional CVD or PVD growth, they have provided important insights that have broadly changed the understanding of NW growth. *In situ* electron microscopy movies of planar NW growth are thus expected to reveal critical details in the mechanism of surface-guided NW growth.

**Figure 1:**
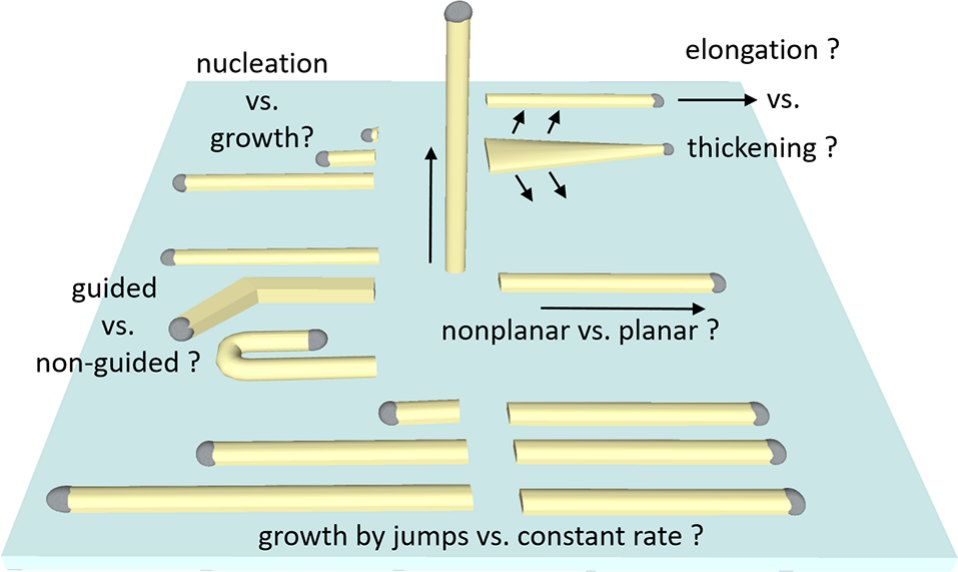
Schematic illustration of different phenomena involved in surface-guided NW growth vs unguided and nonplanar growth, which can be studied by real-time observation during the growth.

Here we observe how surface-guided NWs grow from vapor phase in real-time, using *in situ* SEM. Real-time movies of ZnSe surface-guided NWs on faceted substrates of annealed M-plane sapphire clearly show how the NWs elongate along the substrate nanogrooves while pushing forward the catalytic Au droplet at the tip of the NW. The movies shed light on various important phenomena occurring during the nucleation and growth of the planar NWs (Figure 1), such as planar vs nonplanar growth, catalyst-selective vapor–liquid–solid elongation vs nonselective vapor–solid thickening, the effect of topographic discontinuities of the substrate on the growth direction of the NW, and the elongation of the planar NWs at a constant rate and not by jumps.^46^

## Results and Discussion

The *in situ* study of the surface-guided NW growth was focused on formation of ZnSe NWs on annealed M-plane sapphire, where NWs are known to grow along the nanogrooves of the periodically faceted substrate by the graphoepitaxial mode.^17^ A comprehensive kinetic study on the same material system was published recently, based on *ex situ* data.^34^ The present *in situ* experiments were carried out in a modified SEM, which is especially suited to work under low-vacuum conditions and allows us to introduce gases and evaporate precursor powders without causing damage to the vacuum system or compromising the imaging resolution. We built specially designed heating stages that allow us to heat the precursor powder and the substrate at different temperatures to optimize the growth and imaging conditions. Experiments were performed in two different SEM systems, as detailed in the Methods section and Supporting Information.

ZnSe powder precursor and the annealed M-plane substrate were placed in the heating stage inside the SEM. After pumping down the system, the heating stage was gradually heated to the target temperature in the course of 20 min. When the target temperature was reached, the electron beam was turned on. The imaging parameters (as focus, astigmatism, contrast, brightness, etc.) were quickly adjusted until an image of the surface nanogrooves and Au catalyst nanoparticles became visible. After focusing on the region of growing NWs, the growth process was recorded.

**Figure 2:**
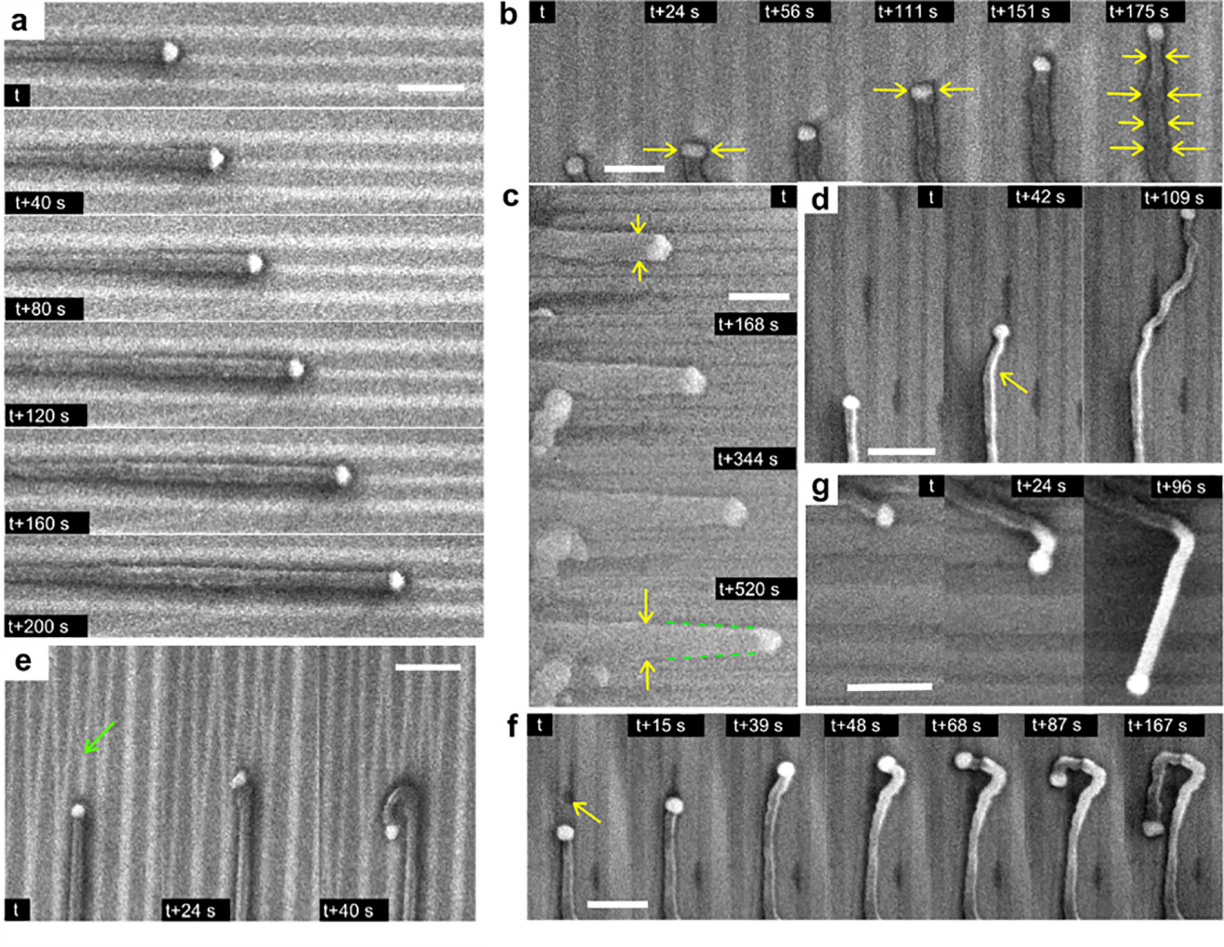
Image sequences of ZnSe NW growth on faceted annealed M-plane sapphire during *insitu* growth inside the SEM. (a) Typical VLS graphoepitaxial guidance where the NW grows directly along the nanogrooves at a constant rate (Movie S1). (b) Changes in the catalyst shape during growth, correlated with a change in the NW thickness (Movie S2). (c) Uncatalyzed vapor–solid growth is occasionally observed on NW sidewalls leading to tapered NWs (Movie S3). Changes in growth direction due to different surface features and effects discussed in the text are demonstrated in (d), (e), and (f) (Movies S4, S1, and S5, respectively). (g) NW changes from planar to nonplanar growth (Movie S6). In these experiments, the substrate temperature was 640 °C, which initiated the highest relative yield of guided in-plane NWs. The temperature of the source powder was kept in the range of 980 to 1020 °C. All scale bars are 500 nm.

Figure 2 shows selected image sequences of ZnSe NW growth on faceted annealed M-plane sapphire during *in situ* growth inside the SEM, representing interesting aspects of the guided NW process. The full movies of all the sequences are available as Supporting Information. Figure 2a (Movie S1, first 7 s) clearly shows the typical VLS mechanism, where the Au-catalyst droplet leads the growth of the NWs at a constant growth rate. The catalyst assists the NW growth by collection and precipitation of material species at the liquid–solid interface. This image sequence also demonstrates the graphoepitaxial guidance mode, as the NW grows directly along the nanogroove.

**Figure 3:**
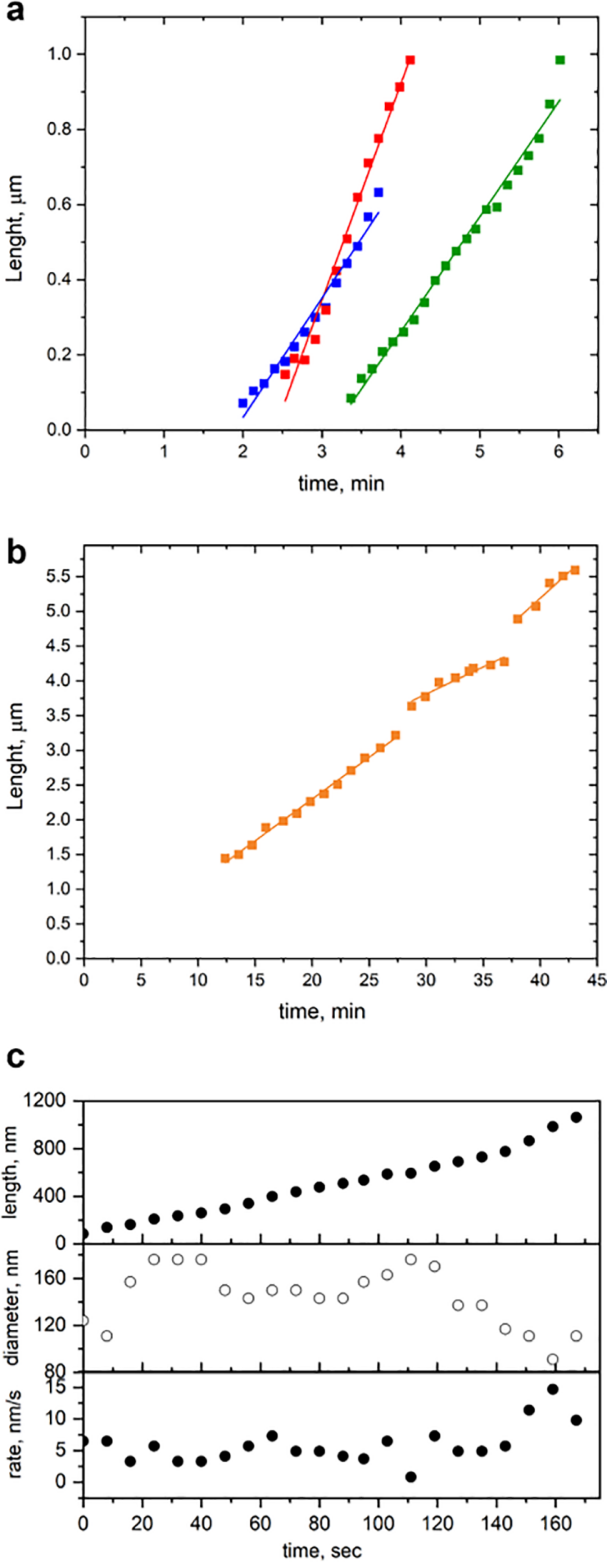
Length vs time of surface-guided NW growth. (a) Growth of three surface-guided NWs, each one of them exhibiting a constant growth rate. The straight lines are linear fits to each data set. (b) Surface-guided NW exhibiting a constant growth rate, even though the NW changed its growth direction twice. (c) Correlation between the droplet diameter and the NW growth rate as measured from the sequence in Figure 2b (Movie S2).

Although the typical VLS growth assumes that the catalyst droplet leads the NW growth and keeps its size and shape constant, the *in situ* observation of guided NW growth shows that the situation is more complex. The sequence of images in Figure 2b (Movie S2) presents changes in the catalyst shape during growth, correlated with the change in the NW thickness. Changon et al. defined it as a combined effect of catalyst surface transport and catalyst dissolving into the NW.^47^ Assuming that the VLS growth process occurs close to the thermodynamic equilibrium (see the discussion of Figure 5), the material flow in/out of the droplet can be locally reversed. Such variations of the material flux result in changing the droplet shape and lead to a change in the width of the NW. These width changes are correlated to the NW growth rate. Figure 3c shows this correlation quantitatively for the specific surface-guided NW shown in Figure 2b. As the growth time proceeds, the diameter of the NW changes, and as a consequence, the growth rate changes as well. An increase in the NW diameter leads to a temporary decrease in the growth rate and *vice versa*. This behavior agrees with the diffusion-mediated part of the growth model,^48^ where slower growth rates are expected for larger NW diameters.

**Figure 4:**
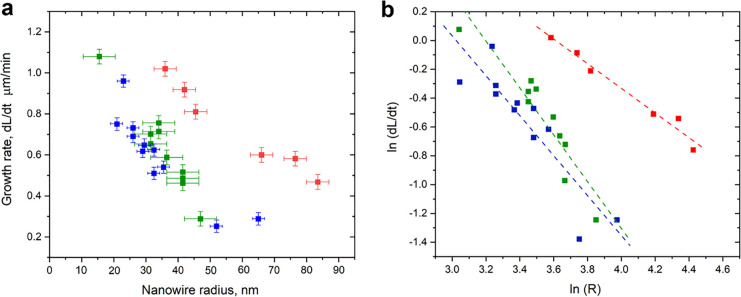
(a) Growth rate vs NW radius for surface-guided NW growth at three different areas (each indicated by a different color) of the annealed M-plane sapphire sample. (b) Scaling analysis of (a); logarithm of the growth rate vs the logarithm of the NW radius. Each dot represents a different NW, and the straight lines represent linear fittings.

**Figure 5:**
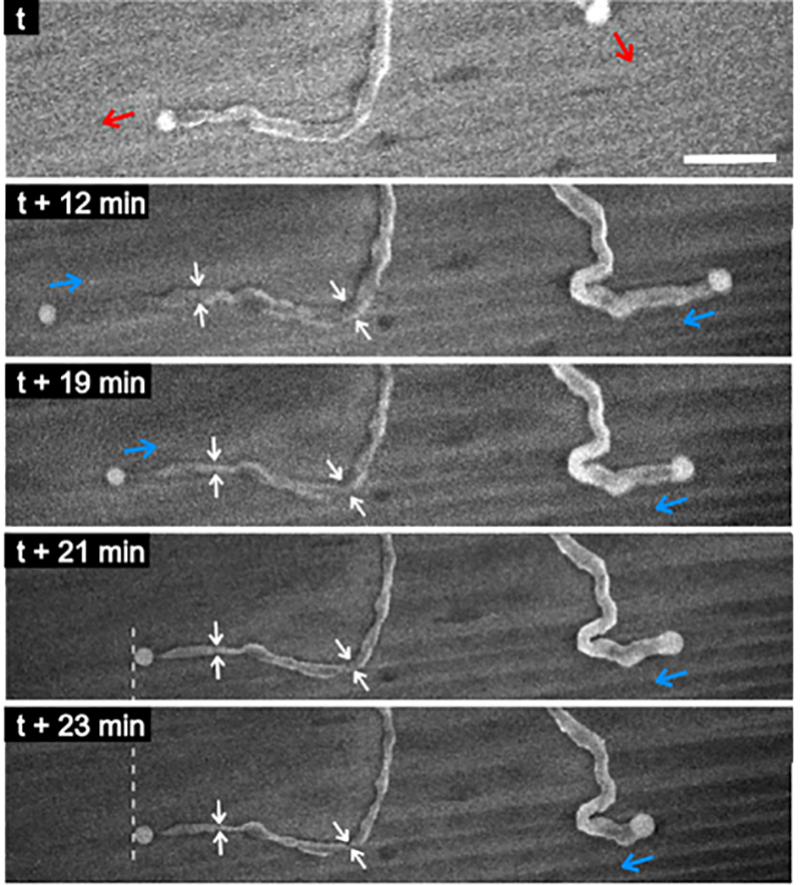
SEM image sequence shows the reverse VLS process, where the NWs shrink back, most probably due to source powder depletion. The arrows indicate the direction of motion of the catalyst nanoparticle; the red arrows indicate growth, whereas the blue ones indicate shrinking. In addition, the white arrows mark the NW cross-section in four consecutive images, clearly indicating NW thinning along its length. Surprisingly, the NW on the left stops shrinking at *t* + 21 min (marked by the white dashed line), obviously due to the vanished LS interface, thus proving the SLV mechanism being behind NW shrinking. Note the NW on the right continues to shrink throughout the whole observation period. The scale bar is 1 μm.

Another process that can lead to changes in the NW diameter is the uncatalyzed deposition of material directly on the NW walls, which increases the NW thickness during the growth (vapor–solid growth, VS). Such a process is shown in Figure 2c (Movie S3). Our *in situ* growth movies show that in this case the surrounding sample surface appears more contaminated by nonselective deposition. This suggests that under certain conditions, perhaps associated with a decrease in the surface-diffusion length, nonselective deposition takes place both on the NW walls and on the substrate. When VS happens at a constant rate simultaneously with VLS, the NWs end up having a tapered shape as in Figure 2c.

In addition to these relatively gradual processes, NWs can abruptly change the growth direction upon different kinds of triggers. In Figure 2d (Movie S4), the planar NW crawls across the nanogroove structure. A similar situation is depicted in Figure 2e (Movie S1, last 6 s), where the guided NW makes a full U-turn when it reaches a region with irregularities in the substrate topography (e.g., merged nanogrooves marked by the green arrow). In Figure 2f (Movie S5) the NW growth path changes and does not follow the original nanogroove. This sudden turn is initiated either by droplet collision with surface contamination (black spot, marked by a yellow arrow) or because of other features not resolved in the SEM image. Thus, the abrupt change in the growth direction and alignment of the NWs can in principle be attributed to different factors, including (1) structural imperfections of the substrate surface, (2) contaminations over the substrate surface, and (3) nonselective deposition of the deposited material on the substrate surface.

As mentioned above, effect (1) can be seen in the growth sequence in Figure 2e, where an imperfection in the periodicity of the substrate nanogrooves is clearly visible and correlated with an NW kink. In this case, several nanogrooves on the growth path of the NW are merged, causing discontinuity of the guiding features, resulting in a loss of the directionality. These imperfections can be caused by an incomplete annealing process of the flat M-plane sapphire. The M-plane sapphire is a thermodynamically unstable plane with a relatively high surface energy.^49^ Upon thermal treatment at elevated temperatures, the M-plane surface undergoes reconstruction and exhibits more thermodynamically stable S-planes and R-planes in periodically faceted V-shaped nanogrooves. While effect (2) is clearly demonstrated in Figure 2f, effect (3) was examined using Auger electron microspectroscopy (Figure S3). The spatially localized analysis has revealed the presence of trace amounts of Zn and Se over the surface in between the NWs.

Besides a change in the direction of planar growth, we sometimes observe a change from planar to nonplanar growth or *vice versa*. For instance, the catalyst droplet may suddenly lose contact with the substrate and keep growing out-of-plane, as presented in Figure 2g (Movie S6). A few TEM analyses (e.g., Figure S4) of such NWs show occasional deposition of elemental Se between the substrate and the NW. This phenomenon (i.e., collision of the guiding droplet with the parasitic Se deposit) could be the origin of a change from planar to nonplanar growth. In other few cases, the NW itself can lose contact with the substrate, while the catalyst remains in contact with the substrate (Movie S7 and the sequence in Figure S5 extracted from it). At a certain moment (between 8 and 16 s), a kink forms, and further NW growth pushes the NW sidewise away from the nanogroove along which it followed before. This raises the question whether all the guided NWs, or all the regions of a guided NW, are covalently bound to the substrate on which they grow. Our observation of well-defined interfaces and epitaxial relations in tens of cross-sectional TEM images from previous studies indicate that guided NWs are usually covalently attached to the substrate.^17^ However, a few movies recorded in our present real-time studies show that some NWs start to grow in a nonplanar mode and only land onto the substrate after reaching a certain length. Subsequently, the growth is switched to a planar mode. Although this is not the common case, one such example is shown in the second part of Movie S7 and in Movie S9.

Another interesting aspect that *in situ* studies allow us to address is the relative timing at which different NWs nucleate and start to elongate. Figure S6 shows an image sequence taken at the initial growth stage. The nucleation events are random and, thus, the resulting NWs have different lengths despite being catalyzed by droplets of a similar diameter. In this respect, in-plane NW growth resembles the out-of-plane growth, where this effect was predicted earlier.^50^ It has been shown that the nucleation can be controlled to some extent by, for example, prefilling the catalyst with one component prior to growth.^34^ This has also been observed for guided ZnSe NWs on sapphire.^17^

Beyond the qualitatively insightful possibility of watching crystal growth on the nanometer scale, analyzing the real-time growth movies of the surface-guided NWs can also provide important quantitative information. Tracking surface-guided NWs on different samples and measuring their actual dimensions as the growth progresses allow us to directly characterize the growth kinetics. For instance, previous studies of the growth mechanism of NWs, both planar and nonplanar, assumed that the growth rate is equal to the postgrowth NW length divided by the growth time (d*L*/d*t* = *L*/*t*). Such an assumption implies that the NW axial growth rate is constant, the nucleation is immediate, and there is no growth termination other than stopping the heating or the precursor feedstock.^51−53^ The *in situ* NW growth experiment allows us to directly measure the length as a function of time *L*(*t*), whose derivative is the instantaneous growth rate d*L*/d*t*, and thus prove if this assumption is correct. Whenever this assumption breaks, this direct observation allows us to correlate the changes in the growth rate with variations in the surface morphology, growth regime (guided vs unguided), or catalyst shape, as will be shown in the next paragraph.

Figure 3 shows length–time plots *L*(*t*) of four representative surface-guided NWs, as derived from two *in situ* NW growth experiments. All the observed NWs exhibit growth rates ranging between 0.21 and 1.10 μm/min. Figure 3a presents data of three surface-guided NWs, monitored for a short time during the growth, showing relatively constant growth rates of 0.31, 0.30, and 0.57 μm/min. The data shown in Figure 3b represent *L*(*t*) for one NW that was monitored and imaged for 45 min during the growth and changed its growth direction twice (at *t* = 28 and 38 min). However, the growth rates for all three growth segments are not completely constant, changing from 0.12 μm/min to 0.08 μm/min and back to 0.13 μm/min. The decrease in the growth rate here may be attributed to the fact that the NW crawls across the nanogrooves, which involves significant changes in the morphology compared to crawling along the nanogrooves.

In a different case, plotted in Figure 3c and visualized in Figure 2b, the growth rate fluctuates within a range of 0–0.9 μm/min on a time scale of approximately 1 min. As shown in the middle graph in Figure 3c, the rate growth fluctuations correlate with variations in the catalyst diameter. A closer look at the images in Figure 2b shows that the catalyst nanoparticle slightly changes its shape from spherical to oblate, back and forth. This suggests that the fluctuations in catalyst diameter are not due to a change in volume, but to oscillating deviations from a hemispherical shape. It is not yet clear whether these oscillations originate from catalyst–NW wetting instabilities, as previously observed in the growth of nonplanar NWs by *in situ* TEM and SEM,^35,52,54^ or else are induced by variations in surface morphology or by catalyst–substrate wetting instabilities.

Next, we study the kinetics of surface-guided ZnSe NWs on annealed M-plane sapphire as a function of nanowire diameter, as we did in our recent report based on *ex situ* measurements,^34^ but here we use the data from real-time measurements by *in situ* SEM, which more accurately represent the instantaneous growth rate d*L*/d*t* than the overall rate growth *L*/*t* estimated from *ex situ* data. For this, several ZnSe NWs were randomly selected in each sample for analysis. The axial growth rates were calculated as the slope of the length vs time plot for each NW. The NW thicknesses were measured directly from the SEM images in the form of projected width. The NW shape was assumed to be 1/2 of a cylinder with radius *R* and thickness 2*R*, on a substrate, according to the simplified growth model.^34^ The droplet guiding its planar growth in a given direction is assumed to be 1/4 of a sphere of the same radius *R*. The NW measured radius is relatively similar to its height, as demonstrated in Figure S7, which allows us to apply the simple geometric model on our experimental data. Even if the projected dimensions measured by SEM are not exactly the thickness of the NW, it is consistently proportional to it (as accurately determined by cross-sectional TEM^17^), so it can serve as an accurate data set for the determination of the power index *m* using our scaling model. The data for three different samples are shown in Figure 4a. Despite some dispersion in the measured data, the NW growth rate clearly correlates with the NWs thickness, as in our previously reported *ex situ* studies. These previous studies revealed two distinct regimes, an increase of the growth rate with the NW width for the thinner NWs (controlled by the GT effect) reaching a maximum at a certain optimal thickness, followed by a long decreasing tail for the thicker NWs (controlled by surface diffusion). However, the trend in the current study points only at a decreasing growth rate as the NW thickness increases. A reason for this is that the present growth experiments were done with Au nanoparticles with a diameter range of 20–150 nm, where growth is limited by surface diffusion rather than by the GT effect. In this regime, it is not necessary to fit the data to the entire eq 1, but only to the diffusion-limited part, which is represented by the second term of the equation, and conveniently simplified as eq 2, where *A* is a constant. This requires only two fitting parameters (*A* and *m*), which enables an accurate determination of the dimensionality of surface diffusion *m*. This turns the problem into a simple scaling analysis, which can be conveniently performed by applying the natural logarithm on eq 2, and plotting ln(d*L*/d*t*) vs ln(*R*) for all the measured NWs, and linear fitting. The linearity of the fitting strongly supports the scaling behavior predicted by the model, and the slope represents the dimensionality of surface diffusion *m*.
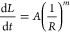
2Figure 4b shows the ln(d*L*/d*t*) vs In(*R*) plots for the same NWs measured in Figure 4a. The *m* values derived from the negative slopes for the red, green, and blue data sets were 0.85 ± 0.07, 1.62 ± 0.23, and 1.38 ± 0.22, respectively. Interestingly, the latter two values, i.e., *m* = 1.62 and 1.38 (green and blue set of data), are very close to 1.5, which is specific for planar growth. As follows from a theoretical analysis,^34^*m* = 1.5 corresponds to the growth regime driven by diffusion across the surface toward the collector droplet. A similar value of *m* = 1.53 ± 0.17 was also observed for the conventional *ex situ* studies on surface-guided ZnSe NWs on C-plane sapphire.^34^ The fitted value of *m* = 0.85 for the red set of data is closer to *m* = 1, which would be more consistent with adatom collection from the upper part of the NW sidewalls, similarly to the nonplanar NWs.^48,55−59^ The discrepancy for this specific data set is not fully understood, but it should be noted that it contains only six NWs, compared to 10 and 11 NWs, respectively, for the first and second sets of data. Another possible origin for the lower scaling in this specific area of the sample might be some degree of surface contamination by amorphous carbon or nonselective deposited material, which could hinder surface diffusion of precursor adatoms on it.

Finally, real-time observation of NW growth also allows us to assess the reversibility of the VLS process on a substrate. It is often presumed that NW growth by the VLS mechanism occurs near thermodynamic equilibrium. Thus, to obtain long, thin, and smooth single-crystal NWs, growers usually tune the reaction conditions by choosing a temperature, a pressure, and the local reactant concentrations that bring the system near equilibrium. A reactive gas (e.g., H_2_) other than the inert carrier gas (e.g., Ar or N_2_) is often added in order to attenuate the growth by reacting with the product back to the reactants following Le Chatelier’s principle. It is difficult to test from *ex situ* experiments whether the assumption that the system is near equilibrium is correct or not. A convincing proof that a system is near equilibrium is obtained when a slight change in the reaction conditions reverses the growth process. A few years after the earlier VLS NW growth papers had been published, the reverse VLS process (referred to as SLV) was discovered and studied.^60−63^ However, it attracted much less attention than the VLS process itself. In this reverse VLS process, the metal droplet was utilized to etch a hole in the crystalline substrate underneath, thus resembling a “negative” NW. In other words, changing the conditions during the reaction resulted in the dissolution of the previously growing NW, leaving no remains but the original metal catalyst droplet.^64^Figure 5 (Movie S8) initially shows planar NW growth led by the catalyst droplet. However, after 16 min of observation during growth, the droplet movement direction is reversed, and the NW starts to shrink back. In addition to shortening, the NW also gets thinner in time. In the end, the thinning process results in diminishing of the solid–liquid interface, leaving the spherical droplet immobile on the surface close to the NW. Based on numerous similar observations of this effect happening after prolonged *in situ* growth, we ascribe it to a decrease in the precursor concentration in the system as the ZnSe source powder is depleted. This precursor concentration decrease shifts the equilibrium toward the reactants, leading to the shrinking of NWs instead of their further growth.

## Summary and Conclusions

We have presented a long-awaited real-time study of surface-guided NW growth. This was demonstrated using *in situ* SEM for planar ZnSe NWs growing along the nanogrooves of a periodically faceted sapphire substrate. The recorded movies allow us not only to visually grasp the process in great detail but also to answer fundamental questions regarding different phenomena that occur during the NW growth. The real-time movies show VLS as the leading mechanism of surface-guided NW growth. The movies demonstrate the graphoepitaxial growth mode along the nanogrooves. They reveal several important aspects of the guided growth mechanism that could not be observed or even suspected from *ex situ* experiments, namely, (i) the effect of surface defects on the guidance of the planar NWs, the timing between competing processes, such as (ii) planar vs nonplanar NW growth and (iii) catalyst-selective VLS elongation vs nonselective VS thickening, (iv) the relatively constant rate of NW elongation, except for (v) slight fluctuations in elongation rate related to changes in the catalyst nanoparticle shape during growth, (vi) confirmation of the two-dimensional scaling of the growth rate with the NW diameter, which the theoretical model attributes to the surface diffusion of precursor adatoms toward the catalyst, and (vii) the reversibility of the growth process, which suggests its occurrence near thermodynamic equilibrium.

Understanding the growth mechanism of surface-guided NWs, and planar NWs in general, has fundamental and practical significance. Introducing the possibility of controlling the NWs’ size and length uniformity by tuning the catalyst size can enable the integration and performance of practical devices. This knowledge may be extended to other material systems with various electronic and optoelectronic applications.

## Methods/Experimental

*In situ* growth experiments were performed in two different SEM systems: (i) The first one is ESEM (Quattro S, Thermo Fisher Scientific), equipped with a 1000 °C heating stage. About 0.05 g of ZnSe powder (American Elements; 99.999%) was loaded into a MgO crucible and placed in the heating stage. A custom-built stainless steel cylindrical cap with a small hole covered the crucible. An annealed M-plane sapphire substrate with a Au catalyst was placed on top of it (Figure S1 Supporting Information). The experiments were performed at 750 °C using a flow of N_2_:H_2_ (95%:5%) gas mixture (Maxima, Ltd.) at a pressure of 200 Pa. The electron energies used were between 15 and 20 keV, and the images were recorded using a gaseous secondary electron detector (GSED) during the NW growth. (ii) A second system utilized in this work comprised a custom-built reactor seated inside an SEM chamber (Quattro, Thermo Fisher Scientific). This concept is similar to the one used in ref (45) and allows us to utilize more detection systems (e.g., in-lens detectors, ET detector), as the microscope chamber can be kept under high vacuum during the experiment (10^–4^–10^–2^ Pa) (Figure S2 Supporting Information). To get closer to the real CVD growth conditions, the reactor utilizes two independent heating stages: one for the sample and the other for vaporizing the solid precursor. The temperature calibration curve (temperature/current passing the heating element) was measured in SEM using temperature-indicating suspensions (Omegalaq) spread on a sample, which allowed us to observe the phase changes associated with a certain temperature in real time with high accuracy (1% according to a manufacturer). This calibration was validated by observing the melting points of different elemental materials. The vapor transport from the precursor heater to the sample is ensured inside the enclosed volume of the reactor by the H_2_ carrier gas injection via a leak valve (up to 2 × 10^–2^ Pa in the main microscope chamber). To avoid the nonselective deposition of precursor on the sapphire substrate, the substrate heater first reaches the target growth temperature (620–650 °C). Only then is the source powder vaporized by the second heater (980–1030 °C). A 30 kV electron beam was used for observation, with beam currents in the range from 50 to 200 pA. The images were acquired with a standard ET detector. Care has been taken to minimize the effect of the electron beam on NW growth. In principle, the beam is capable of activating the surface and increasing the growth rate, or of promoting nonselective VS growth on the exposed surface, leading to formation of larger crystals. To minimize these potential effects, whenever simultaneous nonselective deposition on the edges of the irradiated view field occurred, the electron beam current was decreased (at the expense of lowering the signal-to-noise ratio), which allowed us to obtain reliable and reproducible results.

## Supporting Information Available

Movie S1: VLS graphoepitaxial guidance and change in the NW growth direction (AVI)
Movie S2: changes in the catalyst shape during growth (AVI)
Movie S3: uncatalyzed vapor–solid growth on NW sidewalls (AVI)
Movie S4: changes in NW growth direction due to surface contamination (AVI)
Movie S5: NW growth direction changes due to substrate geometry irregularities (AVI)
Movie S6: NW changes from planar to nonplanar growth (AVI)
Movie S7: NWs lose contact with the substrate while the catalyst remains in contact with the substrate (AVI)
Movie S8: reversibility of the VLS process (AVI)
Movie S9: non-graphoepitaxial growth (MP4)
Movie S10: original uncropped Movie S1 (MP4)
Movie S11: original uncropped Movie S2 (MP4)
Movie S12: original uncropped Movies S4, S5, and S6 (MP4)
Movie S13: original uncropped Movie S3 (MP4)
Movie S14: original uncropped Movie S9 (AVI)
Figures S1 and S2 (reactor configuration), Figure S3 (Auger microanalysis), Figure S4 (cross-sectional TEM analysis), Figure S5 (non-graphoepitaxial NW growth), Figure S6 (*in situ* monitoring of nucleation events), Figure S7 (AFM scan and height analysis of guided NW on sapphire surface) (PDF)

## Supplementary Material

nn2c07480_si_001.avi

nn2c07480_si_002.avi

nn2c07480_si_003.avi

nn2c07480_si_004.avi

nn2c07480_si_005.avi

nn2c07480_si_006.avi

nn2c07480_si_007.avi

nn2c07480_si_008.avi

nn2c07480_si_013.mp4

nn2c07480_si_014.avi
