# Supplementary material for: Real-Time Study of Surface-Guided Nanowire Growth by In Situ Scanning Electron Microscopy
Source: ACS Nano. 2022 Oct 28;16(11):18757–66. doi: 10.1021/acsnano.2c07480 (PMC9706663; doi:10.1021/acsnano.2c07480)
Supplement: Supplementary file 15 — nn2c07480_si_015.pdf [file nn2c07480_si_015.pdf]

Supporting Information

for

Real-Time Study of Surface-Guided Nanowire Growth by *In Situ* Scanning Electron Microscopy

Amnon Rothman*[1], Kristýna Bukvišová*[2,3], Noya Ruth Itzhak[1], Ifat Kaplan-Ashiri[4], Anna Eden Kossoy[4], Xiaomeng Sui[4], Libor Novák[5], Tomáš Šikola[2,3], Miroslav Kolíbal^[2,3] and Ernesto Joselevich^[1]

[1]*Department of Molecular Chemistry and Materials Science, Weizmann Institute of Science, Rehovot 76100, Israel*
[2]*Institute of Physical Engineering, Brno University of Technology, Technická 2, 616 69 Brno, Czech Republic*
[3]*CEITEC BUT, Brno University of Technology, Purkyňova 123, 612 00 Brno, Czech Republic*
[4]*Department of Chemical Research Support, Weizmann Institute of Science, Rehovot 76100, Israel*
[5] *Thermo Fisher Scientific, Vlastimila Pecha 12, 627 00 Brno, Czech Republic*
** contributed equally to this work*
^ corresponding author

Contents:

Movie S1. Typical VLS graphoepitaxial guidance and change in the NW growth direction.

Movie S2. Changes in the catalyst shape during growth.

Movie S3. Uncatalyzed vapor-solid growth on NW sidewalls.

Movie S4. Changes in NW growth direction due to surface contamination.

Movie S5. NW growth direction changes due to substrate geometry irregularities.

Movie S6. NW changes from planar to non-planar growth.

Movie S7. NW lose contact with the substrate while the catalyst remains in contact with the substrate.

Movie S8. Reversibility of the VLS process.

Movie S9. Non-graphoepitaxial growth.

Movie S10. Original uncropped movie of movie S1.

Movie S11. Original uncropped movie of movie S2.

Movie S12. Original uncropped movie of movies S4, S5 and S6.

Movie S13. Original uncropped movie of movies S3.

Movie S14. Original uncropped movie of movies S9.

Figure S1. The first reactor configuration within the Quattro S ESEM.

Figure S2. The second reactor configuration within the Quattro -SEM.

Figure S3. Auger microanalysis of surface in between the NWs.

Figure S4. Cross-sectional TEM analysis of the NWs

Figure S5. Non-graphoepitaxial NW growth (image sequence extracted from movie S7)

Figure S6. *In situ* monitoring of the nucleation events.

Figure S7. AFM scan and height analysis of guided NW on sapphire surface.

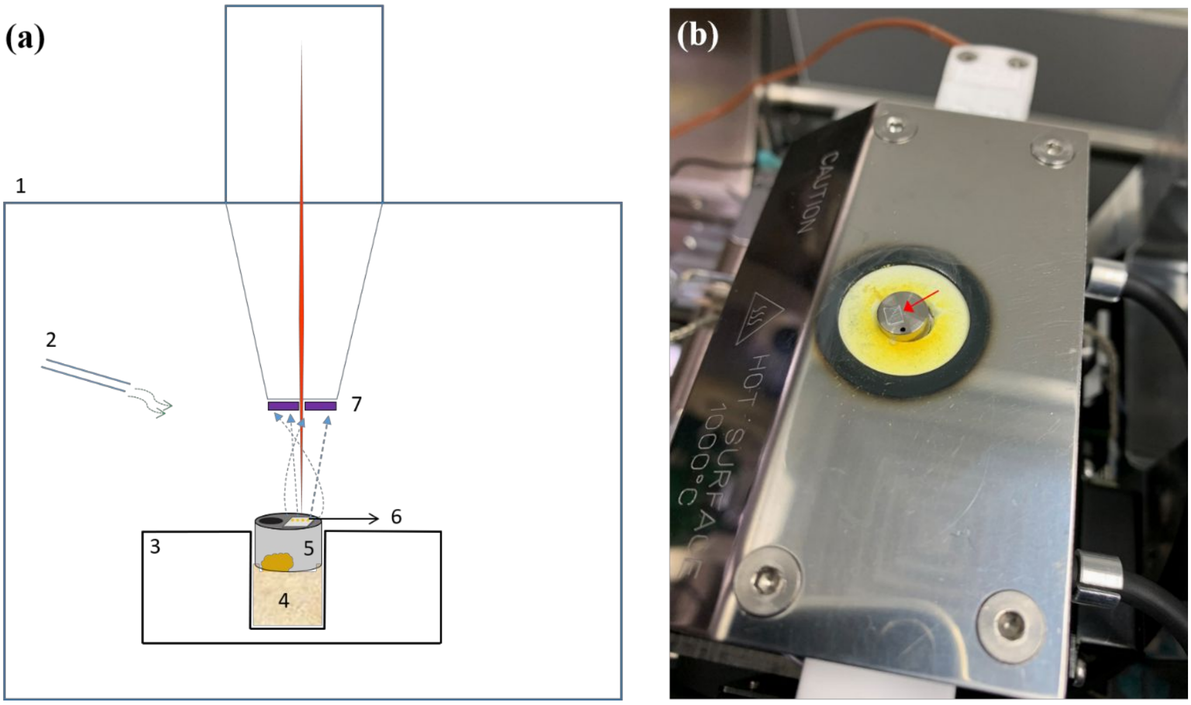

Figure S1. The first reaction configuration within the Quattro S (Thermo Fisher Scientific) ESEM. (a) Schematics of (1) the ESEM chamber, (2) N_2/H_2 gas inlet and the (3) heating stage. Inside the heating stage a (4) MgO crucible was placed and the precursor powder was loaded the heated cavity, covered with (5) homemade stainless steel cylindrical shield. The substrate (6) was placed on top of the cylindrical shield and the secondary electron signal was collected with the detector. (b) Real top-view image of the heating stage. The red arrow points on the sapphire substrate on top of the stainless-steel cover.

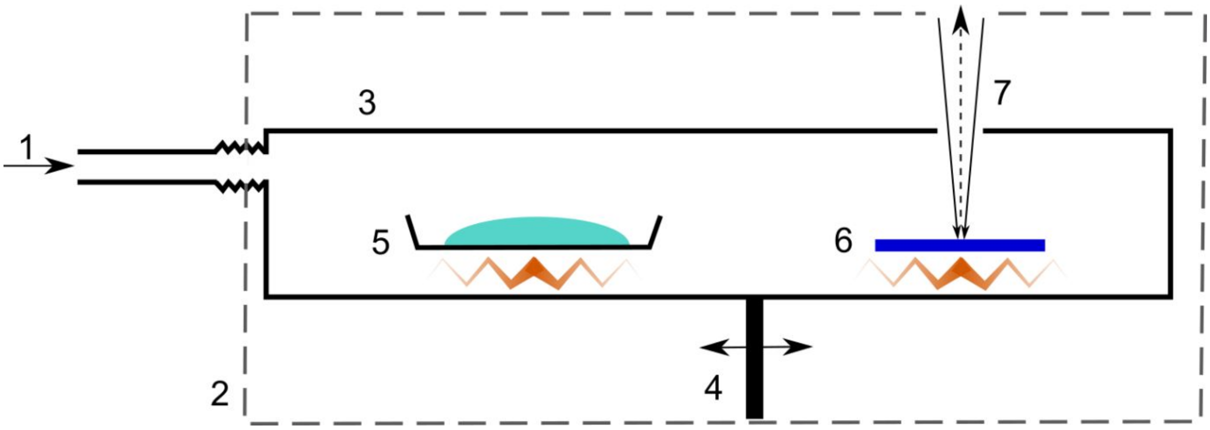

Figure S2. The second reaction configuration within the Quattro (Thermo Fisher Scientific) SEM (2). A gas inlet (1) serves as an entrance of a carrier gas (H_2) into the reactor (3). The reactor is mounted on a sample stage (4) and incorporates two heater stages: precursor heater (5) and substrate heater (6), which are controlled separately and both allow to reach temperatures up to 1000 °C. Both heaters are controlled by thermocouples and were calibrated utilizing the melting points of several elements, covering the entire working temperatures window. The reactor is not sealed; the pressure inside does not exceed $5{\cdot}10^{-2}$ Pa, also because 8 mm^2 large aperture (7) that allows to observe the processes on the sample. Detection is done using standard Everhart-Thornley detector in SE mode.

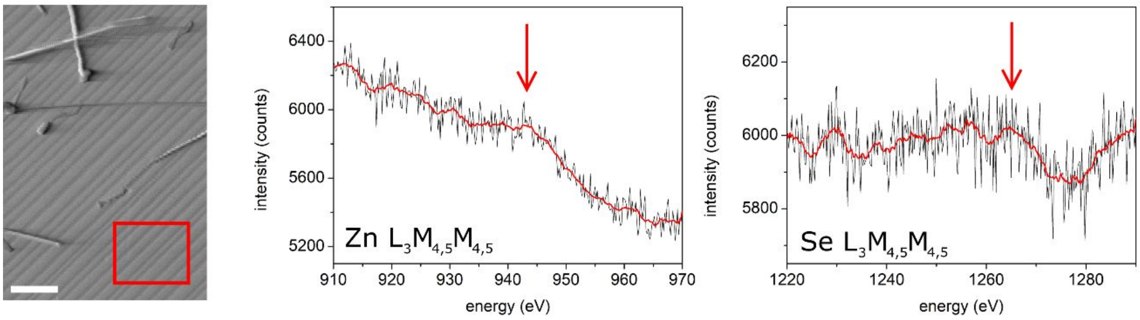

Figure S3. Auger microanalysis of surface in between the NWs. The image on the left shows an analysis area, which comprises seldom ZnSe nanowires (both in-plane and out-of-plane), and mostly free surface in between. Red rectangle marks the area where Auger microanalysis has been carried out. Two regions of the spectrum are shown – Zn $L_3M_{4,5}M_{4,5}$ and Se $L_3M_{4,5}M_{4,5}$. The red arrows mark the position of most intensive transitions according to the element libraries. Clearly, Zn and Se can be detected even on a bare surface of the sample. Note that despite being 'invisible' to the secondary electron image, the uncatalyzed deposit is identified from Auger spectrum, even without the need to differentiate the spectrum, which is a common practice. The spectra were acquired utilizing Omicron Nanosam Auger electron microscope, utilizing beam current of 1 nA and 3 keV primary energy. The vacuum conditions within the microscope are below 5.10^{-10} mbar. Scale bar in the left image is 1 μm.

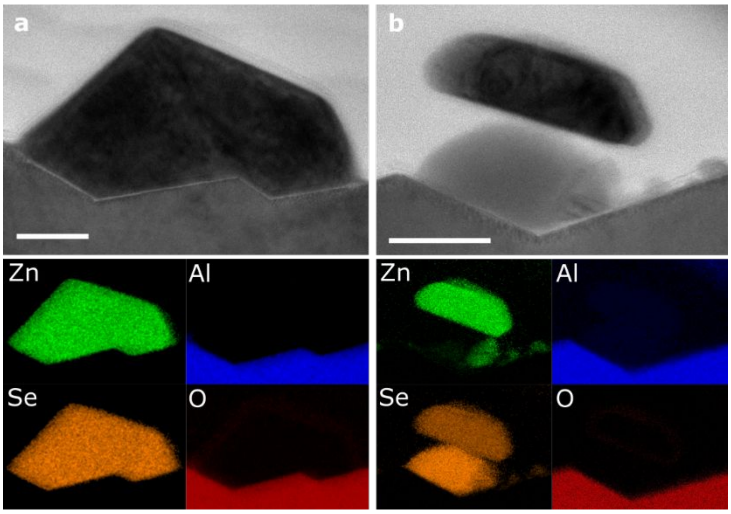

Figure S4. The TEM images and EDX maps show (a) graphoepitaxial relationship between nanowires and the substrate, but also interfacial layers found at this interface in case of irregular nanowires. (b) An example of deposition of elemental Se between the substrate and the NW, could be the origin of a change from planar to nonplanar growth. Both Zn and Se are identified and mapped by EDX. Scale bar is 50 nm.

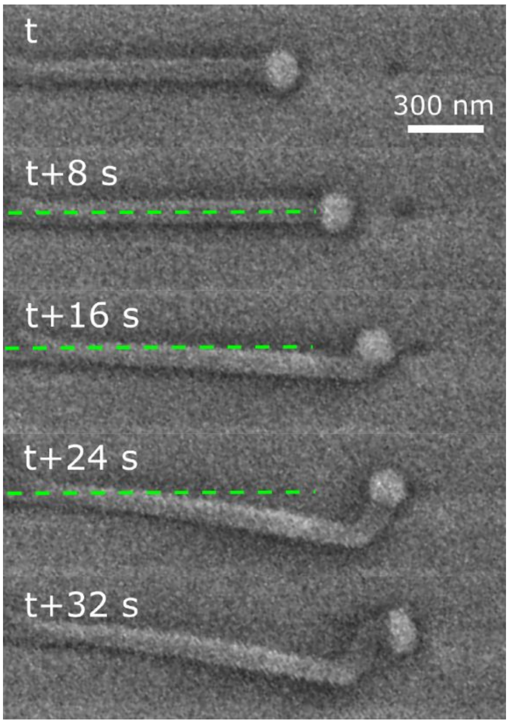

Figure S5. Non-graphoepitaxial NW growth (image sequence extracted from movie S7). Although growing seemingly within the groove, at t+16 s the guiding droplet changes movement direction (away from the groove axis marked by the green line) and the resulting force pushes the nanowire outside the groove. Such behavior is possible only if the interaction between the nanowire and the substrate is very weak or none. In the latter case, the droplet is only guiding the nanowire growth within the groove, and the graphoepitaxy is not established. Interestingly, the nanowire contrast gets brighter after the movement. We ascribe this change to electron-beam-induced charging caused by the fact that the NW segment is not in contact with the substrate anymore. The rest of the movie S7 (not in sequence) shows that the subsequent NW growth can continue within the groove again after two loops are formed and the droplet (with the near NW segment) falls down to the groove. This interpretation is based on the contrast changes observed on the NW (brighter – nanowire is out-of-plane, darker – nanowire is in-plane). The substrate temperature was 640 °C, the temperature of the source powder was kept in the range of 980 °C to 1020 °C.

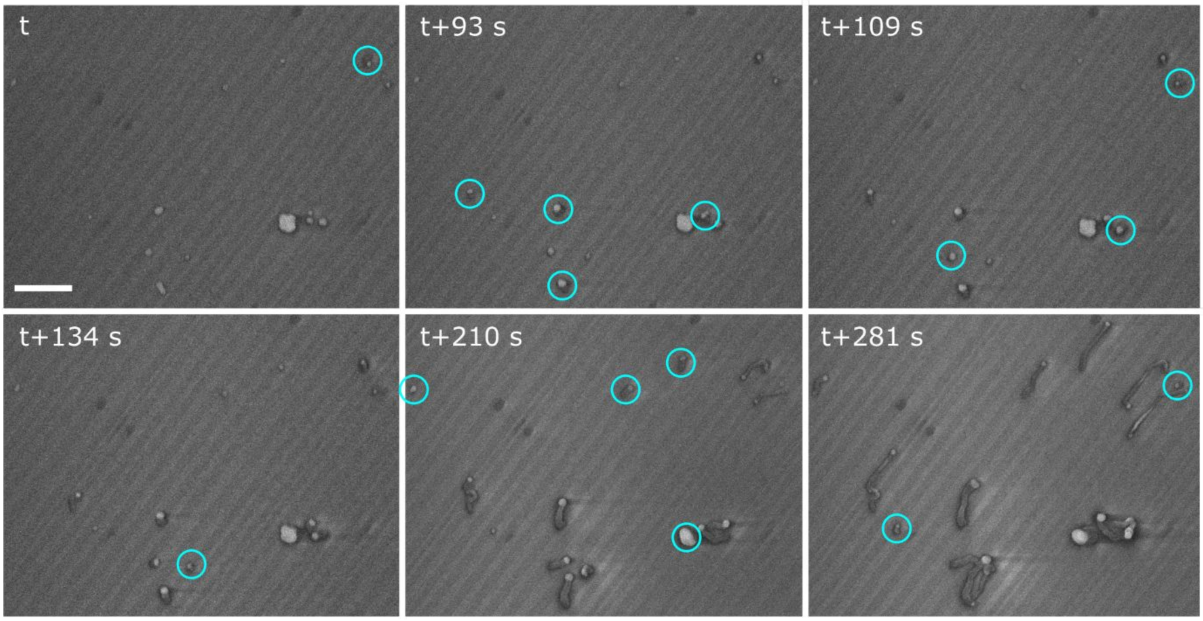

Figure S6. Monitoring the nucleation events in the initial period of the growth. Turquoise circles mark the droplet(s) from which the nanowire just started to grow. The nucleation events are random and there is no traceable dependence of nucleation time on the catalyst's size. Substrate temperature was 640 °C and source powder was kept in the range 1000 °C to 1020 °C. Scale bar is 1 µm.

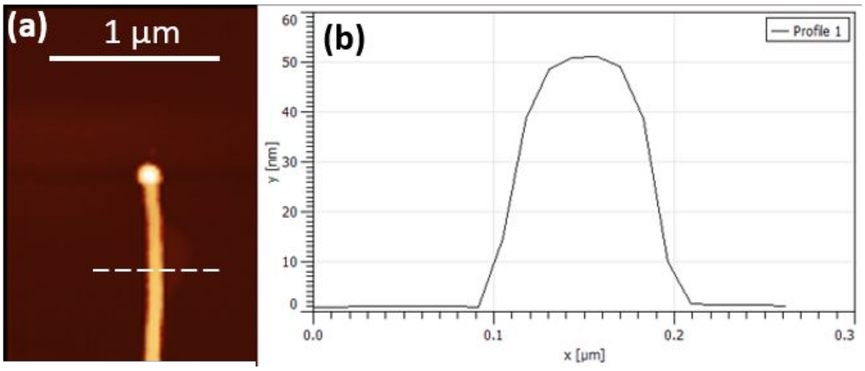

Figure S7. (a) AFM scan of guided NW on sapphire surface. (b) Height profile across the dashed line in (a). The measured width of the NW, which equals to 2R is ~120 nm, hence R~60 nm. The measured height of the NW is ~50 nm. The half-width-to-height ratio is 1.2.
